# First preclinical experience with the newly developed EDGE SP1000 single-port robotic surgical system-assisted transanal total mesorectal excision

**DOI:** 10.1093/gastro/goab039

**Published:** 2021-10-28

**Authors:** Liang Kang, Hua-Shan Liu, Zi-Wei Zeng, Shuang-Ling Luo, Xing-Wei Zhang, Liang Huang, Jian-Chen Wang, Ping Lan

**Affiliations:** 1 Department of Colorectal Surgery, The Sixth Affiliated Hospital of Sun Yat-sen University, Guangzhou, Guangdong, P. R. China; 2 Guangdong Provincial Key Laboratory of Colorectal and Pelvic Floor Diseases, The Sixth Affiliated Hospital of Sun Yat-sen University, Guangzhou, Guangdong, P. R. China; 3 Shenzhen Jingfeng Medical Technology Co., Ltd, Shenzhen, Guangdong, P. R. China

## Introduction

With advances in technology, natural orifice transluminal endoscopic surgery (NOTES) has long been an ultimate clinical pursuit in minimally invasive surgery. The transanal total mesorectal excision (taTME) in rectal surgery has been considered a representative example of the adoption of the NOTES concept [1, 2]. The taTME procedures offer an optimized view, better exposure of the anatomical plane, more accurate identification of the resection margin in the narrow pelvis, as well as a more direct approach to the most problematic aspect of the distal rectal dissection [3, 4]. However, traditional taTME using laparoscopic instruments has residual limitations regarding the technical hurdles related to limited maneuverability and a steep learning curve due to the very distinct anatomical landmarks relative to the conventional surgical approach. These largely impede progress in the clinical practice of taTME.

The single-port (SP) multichannel robotic system provides an enhanced visualization of the surgical field and better maneuverability of the instruments with optimal stability. This enables ambidextrous movements to be achieved, decreases tremor, and improves dexterity, thus allowing a more precise dissection even in a confined space. These facets make SP robotics ideally suitable for taTME procedures. The adoption of the da Vinci SP system (Intuitive Surgical, Inc., CA, USA) in the taTME procedures has gained considerable interest. Kneist *et al.* [5] demonstrated the technical feasibility of the SP robot-assisted taTME in a cadaveric model. The daunting challenges of laparoscopic taTME, especially the long learning curve and limited maneuverability, are overridden by the robotics, thereby making taTME much easier to learn and adopt. However, the da Vinci SP platform hitherto remains the only system approved for clinical use. Worse yet, due to the low installed capacity and high medical costs of the da Vinci SP system, only a few individuals have the fortune of experiencing the system. Despite the encouraging benefits, these constraints have seriously hampered the advancement and widespread application of SP robotic techniques in taTME.

**Figure 1.**
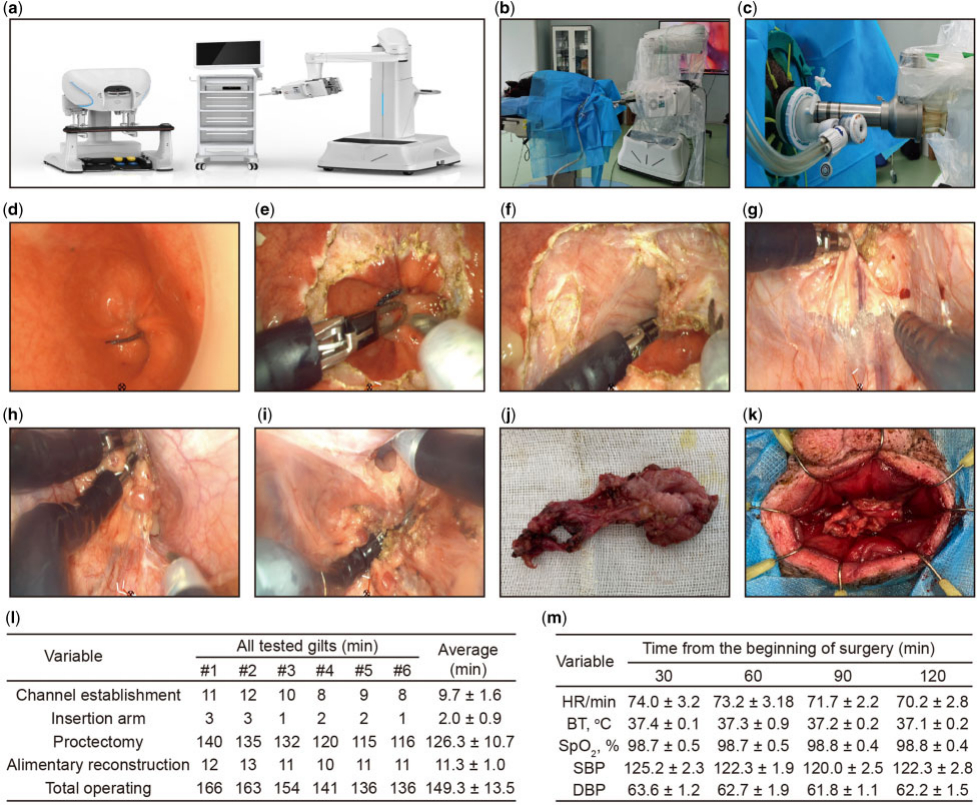
Key steps of EDGE SP1000 single-port robotic surgical system-assisted taTME in porcine model. (A) EDGE SP1000 single-port robotic surgical system (Shenzhen Jingfeng Medical Technology Co., Ltd, Shenzhen, China). (B) The whole scene of the robotic surgical system-assisted taTME. (C) Installation of single-port channel. (D) Closing the intestinal lumen using purse-string suture. (E) Circumferential dissection of the rectal mucosa. (F) Circumferential dissection of the rectal muscle layer. (G) Mesorectal excision (behind). (H) Mesorectal excision (lateral). (I) Dissection of peritoneal reflection (ahead). (J) The total mesorectal excision specimen. (K) An end-to-end straight stapled hand-sewn anastomosis. (L) The time of each stage of the operation; average is presented as mean ± standard deviation. (M) Intraoperative vital-signs monitoring records. All data are presented as mean ± standard deviation. HR, heart rate; BT, body temperature; SpO_2_, saturation of pulse oximetry; SBP, systolic blood pressure; DBP, diastolic blood pressure; taTME, transanal total mesorectal excision.

Against this background, the novel EDGE SP1000 single-port robotic surgical system (Edge Medical Robotics Co., Ltd, Shenzhen, China) was recently designed and constructed by the authors who own fully independent intellectual-property rights. The EDGE SP1000 system is based on the perfect combination of single-port laparoscopic technology and a robotic surgery system (**Figure 1A**). With good performance in various tests, it was approved by the Beijing Institute of Medical Device Testing, National Medical Products Administration in December 2020. Liu *et al.* [6] have demonstrated their experience with this newly developed robot, revealing its feasibility in nephrectomy. Herein, we present our first experience with the newly developed EDGE SP1000 single-port robotic surgical system-assisted taTME in a porcine model. This work evaluated the feasibility of our newly developed robot in taTME by testing the intraoperative information including vital signs, surgical complications, and the survival outcomes of test gilts for a 2-week follow-up period.

## Materials and methods

The study was approved by the institution of Animal Care and Use Committee of Songshan Lake Pearl Laboratory Animal Science and Technology Co., Ltd, Dongguan, Guangdong, China (IACUC: QR-MR003-002-A0). A total of six female gilts (weighing 35–40 kg and aged 11–13 months) that were offered by Songshan Lake Pearl Laboratory Animal Science and Technology Co., Ltd, underwent the EDGE SP1000 single-port robotic surgical system-assisted taTME. The taTME procedures were mainly performed as follows (**Supplementary Video 1**) (i) Before the operation, the supply of food and drink to the sample gilts was withdrawn for 12 and 6 h, respectively. (ii) After anesthesia, the gilts were fixed in a supine position (**Figure 1B**). (iii) Following disinfection of the perineum and anus of the gilts and anal dilation, the anorectum was sufficiently exposed using the anal retractor system. (iv) Under direct vision, the purse string was sutured to close the intestinal lumen about 3 cm from the anus using a semicircular anal speculum (**Figure 1D**). (v) Before incising the rectal wall, the lavage lumen was washed using 500 mL iodophor and 500 mL saline. (vi) The EDGE SP1000 single-port was introduced into the anal cavity through the Gel-port (AppliedMedical, Rancho Santa Margarita, CA, USA) (**Figure 1C**). (vii) Subsequently, the Airseal iFS (ConmedTM, Utica, NY, USA) was adopted to create a pneumo-anorectum (12–15 mmHg). (viii) After the introduction of EDGE SP1000 instruments including a camera and grasper via the port, a full-thickness circumferential dissection was performed until reaching the perirectal plane (**Figure 1E-I**). (ix) The rectum was pulled out through the anus and the specimen was transected and removed (**Figure 1J**). (x) Following the withdrawal of the robotic system, an end-to-end hand-sewn anastomosis was performed under direct vision using 2–0 Vicryl (**Figure 1K**).

The study was approved by the institution of Animal Care and Use Committee of Songshan Lake Pearl Laboratory Animal Science and Technology Co., Ltd (Shenzhen, China) (IACUC: QR-MR003-002-A0).

## Results

The mean time for channel establishment, EDGE SP 1000 arm insertion, proctectomy, alimentary reconstruction, and total operating time was 9.7 ± 1.6, 2.0 ± 0.9, 126.3 ± 10.7, 11.3 ± 1.0 and 149.3 ± 13.5 min, respectively (**Figure 1L**). Stable vital signs were obtained for all six gilts during the procedures (**Figure 1M**). Marked operational problems and hurdles were not encountered during the operation of the EDGE SP1000 single-port robotic system and no significant intraoperative adverse events occurred during surgery. Within 1 day after the operation, no tested gilts displayed significant trembling and moaning behavior. All tested gilts exhibited good recovery within 3 days after the operation, with normal mental state, bowel function, free movement, as well as healthy drinking and eating activity. Within 2 weeks after surgery, no gilts experienced post-operative complications before they were sacrificed under anesthesia. Taken together, these findings indicated the feasibility of robotic taTME using our newly developed EDGE SP1000 single-port robotic surgical system.

## Discussion

A pioneering study by Verheijen *et al.* [7] clinically explored robotic single-site taTME using the da Vinci multiport robotic surgical system. However, such a system exhibited remarkable weaknesses including loss of triangulation and external collisions between instruments. This scene led to crowded operative space, additional challenges in intracorporeal traction, suturing, and operative time extension. In contrast, our newly developed EDGE SP1000 single-port robotic system shows unique benefits for taTME operation. Based on our experience from porcine models, taTME can be much more easily conducted through our SP robotic platform. Featuring an ideal combination of a flexible camera manipulator arm and three wrist-type manipulator arms, the EDGE SP1000 system has no intersection of the robot arms, avoiding mutual interference between the instruments even in narrow operating spaces. Under the assistance of the camera system, it is much easier for surgeons to identify the nerves, capillaries, fascia, and other tissues. Due to its wrist-type manipulator arms, surgeons are able to achieve a cross-operation mode in which “the right hand does the work on the left and the left hand does the work on the right.” Moreover, hand physiological tremor of surgeons could be entirely eliminated using the EDGE SP1000 robotic system, offering more flexible and accurate taTME procedures.

Our robotic system can be used to operate taTME in an animal model. Besides, we consider these characteristics of our SP robotics conducive to overcoming the technical difficulties and steep learning curve associated with the complexity of taTME. Following the NOTES concept and the remarkable advantages of robotic taTME, our positive findings support the introduction of our robotic system into clinical practice.

## Authors’ Contributions

L.K. and P.L. were in charge of project development and were chief surgeons of the robotic taTME. H.S.L. and Z.W.Z. were assistants for the taTME surgery. S.L.L., X.W.Z., and J.C.W. were responsible for data collection or management. H.S.L. and Z.W.Z. performed data analysis. H.S.L. and Z.W.Z. did article writing. L.K. and P.L. did article editing. All authors read and approved the final manuscript.

## Funding

This project was supported by grants from the Fundamental Research Funds for the Central Universities [grant number 16ykjc25], National Key R&D Program of China [grant number 2017YFC1308800], and the Sun Yat-sen University Clinical Research 5010 Program [grant number 2016005].

## Supplementary Material

goab039_supplementary_data

## References

[1] Sylla P , Rattner DW, Delgado S et al NOTES transanal rectal cancer resection using transanal endoscopic microsurgery and laparoscopic assistance. Surg Endosc 2010;24:1205–10. doi:10.1007/s00464-010-0965-6. 20186432 10.1007/s00464-010-0965-6

[2] Kang L , Sylla P, Atallah S et al taTME: boom or bust? Gastroenterol Rep (Oxf) 2020;8:1–4. doi:10.1093/gastro/goaa001. 32104580 10.1093/gastro/goaa001 PMC7034229

[3] Kang L , Chen YG, Zhang H et al Transanal total mesorectal excision for rectal cancer: a multicentric cohort study. Gastroenterol Rep (Oxf) 2020;8:36–41. doi:10.1093/gastro/goz049. 32104584 10.1093/gastro/goz049 PMC7034231

[4] Kang L , Zeng Z, Luo S et al Transanal vs laparoscopic total mesorectal excision for rectal cancer: a multicenter randomized phase III clinical trial (TaLaR trial) protocol. Gastroenterol Rep (Oxf) 2021;9:71–6. doi:10.1093/gastro/goaa083. 33747528 10.1093/gastro/goaa083 PMC7962745

[5] Kneist W , Stein H, Rheinwald M. Da Vinci single-port robot-assisted transanal mesorectal excision: a promising preclinical experience. Surg Endosc 2020;34:3232–5. doi:10.1007/s00464-020-07444-4. 32394173 10.1007/s00464-020-07444-4 PMC7271049

[6] Liu C , Lai C, Yao X et al Robot-assisted nephrectomy using the newly developed EDGE SP1000 single-port robotic surgical system: a feasibility study in porcine model. J Endourol 2020;34:1149–54. doi:10.1089/end.2020.0208. 32911971 10.1089/end.2020.0208

[7] Verheijen PM , Consten EC, Broeders IA. Robotic transanal total mesorectal excision for rectal cancer: experience with a first case. Int J Med Robot 2014;10:423–6. doi:10.1002/rcs.1594. 24807675 10.1002/rcs.1594

